# Therapeutic Potential of Cytoglobin and Neuroglobin in Oxidative Stress-Driven Liver Diseases

**DOI:** 10.3390/antiox15040485

**Published:** 2026-04-14

**Authors:** Le Thi Thanh Thuy, Hoang Hai, Pham Tuan Anh, Nguyen Bui Tam Chi, Tran Van Bao, Tran Dang Anh Huyen, Nguyen Tran Quang Sang, Michelle L. Hermiston

**Affiliations:** 1 Department of Global Education and Medical Sciences, Graduate School of Medicine, Osaka Metropolitan University, Osaka 545-8585, Japan; 2 Sarcoma Center, Vinmec Times City International Hospital, Vinmec Healthcare System, Hanoi 11622, Vietnam; 3 Center for Innovation in Health Sciences, College of Health Sciences, VinUniversity, Hanoi 12400, Vietnam

**Keywords:** globin, antioxidant, liver cancer, liver fibrosis

## Abstract

Chronic liver diseases, including fibrosis and hepatocellular carcinoma (HCC), are primarily driven by oxidative stress, yet traditional antioxidant therapies often lack the specificity and efficacy required for clinical success. This review evaluates the emerging therapeutic potential of two atypical globins, cytoglobin (CYGB) and neuroglobin (NGB), exploring their unique hexacoordinated heme structures that enable potent reactive oxygen and nitrogen species (ROS/RNS) scavenging and redox-regulated signaling. We summarize a broad range of in vitro and in vivo evidence demonstrating that these globins deactivate hepatic stellate cells, reduce extracellular matrix accumulation, and function as tumor suppressors by modulating pathways such as Raf/MEK/ERK and NRF2. In human cohorts, CYGB expression levels inversely correlate with the progression of Metabolic Dysfunction-Associated Steatohepatitis (MASH) and HCC, highlighting its potential as a clinical biomarker. Furthermore, recombinant protein therapies involving CYGB and NGB show promise in promoting collagen degradation and inhibiting malignant transformation. We conclude that CYGB and NGB represent sophisticated catalytic redox regulators that offer a novel therapeutic paradigm for restoring redox homeostasis. While delivery and pharmacokinetic barriers remain, these globins are highly promising candidates for first-in-class biologics in hepatology.

## 1. Introduction

The liver serves as the body’s primary metabolic and detoxifying hub, making it a central target for various insults, including viral infections, alcohol abuse, and metabolic dysfunction. Oxidative stress not only implicated in acute liver injury and in the pathogenesis but also is a critical and convergent driver in the pathogenesis of chronic liver diseases such as fibrosis, cirrhosis, and HCC [1]. Oxidative stress arises from an imbalance between the production of reactive oxygen and nitrogen species (ROS/RNS) and the capacity of endogenous antioxidant systems to neutralize them. Although the body possesses a complex antioxidant network, these systems often face therapeutic gaps and limitations in response to chronic injury.

**Figure 1:**
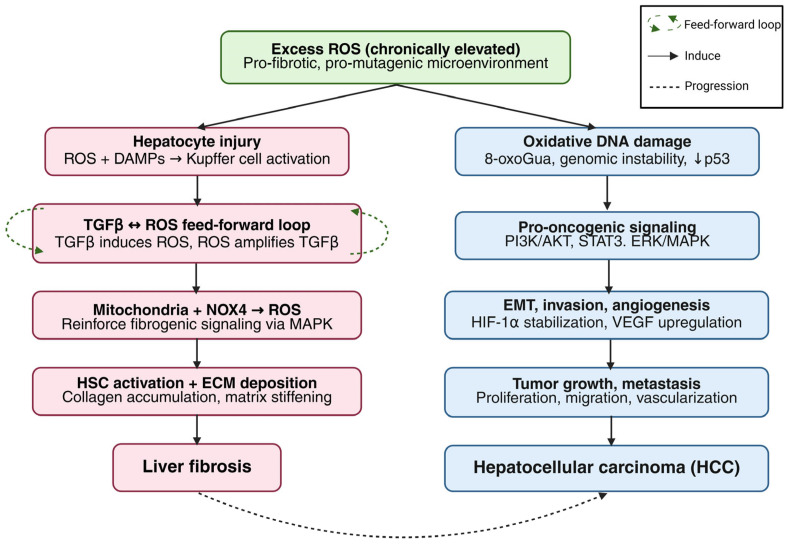
Role of ROS in liver fibrosis and hepatocellular carcinoma (HCC). Chronic ROS accumulation induces DNA damage, lipid peroxidation, and protein oxidation. These events collectively activate TGF-β-mediated fibrogenesis and pro-oncogenic signaling (ERK/AKT/STAT3, HIF-1α/VEGF), ultimately driving HCC development. Created in BioRender. Education, G. (2026) https://BioRender.com/lba5kqc, accessed on 5 April 2026.

When chronically elevated, reactive oxygen species (ROS) serve as central pathogenic drivers of liver fibrosis and hepatocellular carcinoma (HCC) by directly activating hepatic stellate cells to orchestrate a pro-fibrotic niche, while simultaneously fostering a pro-mutagenic tumor microenvironment through oxidative DNA damage, genomic instability, and epithelial–mesenchymal transition [1,2]. In the context of liver fibrosis, ROS regulate hepatocyte injury, inflammatory signaling, and the activation of hepatic stellate cells (HSCs) [3]. Injured hepatocytes release both ROS and damage-associated molecular patterns (DAMPs) that trigger the activation of Kupffer cells and HSCs. This process is further sustained by a self-amplifying TGF-β–ROS feed-forward loop that drives continuous HSC activation and subsequent extracellular matrix (ECM) deposition [3]. ROS originating from mitochondria and NADPH oxidases—particularly NOX4—reinforce these fibrogenic signals, primarily through the activation of MAPK pathways. In the progression toward HCC, ROS facilitate tumor development by inducing oxidative DNA damage, such as 8-oxoguanine formation, which promotes genomic instability and mutagenesis [4]. Furthermore, ROS disrupt the function of critical tumor suppressors like p53 while simultaneously activating pro-oncogenic pathways, including PI3K/AKT, STAT3, and ERK/MAPK. Additionally, ROS promote the epithelial–mesenchymal transition (EMT), tumor invasion, and angiogenesis by stabilizing HIF-1α and upregulating VEGF expression (Figure 1, [3,4]).

Liver fibrosis involves a complex interplay of inflammatory and immune cells, with redox reactions playing a pivotal role in activating the hepatic myofibroblasts responsible for the overproduction of collagen and ECM proteins [5]. CYGB, first identified in HSCs in 2001, possesses a robust capacity for free radical scavenging [6]. Due to its unique structural conformation, CYGB acts as an intrinsic regulator of redox homeostasis, establishing it as a highly promising therapeutic candidate and a focal point of intensive global research.

Experimental evidence from Cygb-deficient murine models has comprehensively demonstrated that the absence of this protein exacerbates oxidative stress, hepatic fibrogenesis, and hepatocarcinogenesis in various contexts, including diethylnitrosamine (DEN) administration, diet-induced steatohepatitis, physiological aging, and bile duct ligation-induced cholestasis [7]. Conversely, these pathological manifestations are markedly attenuated in Cygb-overexpressing transgenic mice [8,9]. Furthermore, a Hexa His-tagged recombinant human CYGB (His-CYGB) has been engineered and validated for its superior ROS-scavenging efficacy. Intravenous administration of His-CYGB has been shown to successfully target murine HSCs, effectively mitigating fibrosis progression in models induced by thioacetamide (TAA) or 3,5-diethoxycarbonyl-1,4-dihydrocollidine (DDC) [9]. This established anti-fibrotic capacity provides a compelling rationale for investigating other members of the globin superfamily—specifically hemoglobin (HB), myoglobin (MB), and NGB [10].

Among these structural homologs, neuroglobin (NGB) stands out as a compelling candidate due to its profound capacity to regulate cellular redox homeostasis. First identified in 2000 by Burmester et al., NGB was reported to be predominantly expressed in the human brain, with the strongest signals observed in the frontal lobe, subthalamic nucleus, thalamus, and murine neurons [11]. Beyond the nervous system, NGB expression has been documented across various normal human tissues, including the liver, kidney, and pancreas, as well as in human glioblastoma multiforme cell lines [12].

Functionally, NGB serves as a physiological sensor for hypoxia and oxidative stress. Research has shown that oxidized NGB responds to redox imbalances by binding to the GDP-bound G-alpha subunit, acting as a guanine nucleotide dissociation inhibitor [13]. This redox-sensing ability relies on an intramolecular disulfide bond between the Cys46 and Cys55 residues [14]. NGB levels increase significantly during hypoxia or when triggered by hypoxia-mimetic agents like cobalt or deferoxamine [15]. Under severe oxidative stress, NGB translocates to the mitochondria to interact with cytochrome C, thereby blocking apoptotic initiation [16,17].

NGB also functions as a redox-regulated nitrite reductase, converting nitrite into nitric oxide (NO). The rate of this reaction depends on the heme coordination state, which is modulated by surface cysteine residues; transitioning to a five-coordinate geometry significantly accelerates NO production, which subsequently inhibits cellular respiration by targeting cytochrome c oxidase [14]. Beyond sensing, NGB acts as a potent antioxidant. Recombinant human NGB (rhNGB) neutralizes various ROS, such as superoxide anions and hydrogen peroxide, with an efficiency comparable to Vitamin C. Unlike HB, rhNGB also possesses unique iron (Fe^2+^) chelating activity, confirming its intrinsic cytoprotective potential for treating oxidative stress-driven diseases [18].

The necessity of harnessing advanced redox modulators is underscored by current challenges in hepatology. While the landscape for chronic liver diseases like MASH has evolved with metabolic-targeted therapies—such as the FDA-approved THR-β agonist Resmetirom—a critical gap remains [19,20]. Traditional non-enzymatic antioxidants like Vitamin E or C often show inconsistent clinical success due to poor tissue delivery and a lack of specificity [21].

Therefore, beyond metabolic therapies and broad-spectrum antioxidants, there is a critical need for targeted strategies that achieve the redox-mediated deactivation of HSCs, the primary effectors of ECM deposition [22]. Unlike traditional globins (HB and MB) that primarily transport or store oxygen, atypical globins like CYGB and NGB function as active, catalytic redox sensors. Their unique structural features, such as hexacoordinate heme iron, enhance their ability to participate in redox reactions and scavenge harmful radicals, offering a novel therapeutic paradigm for restoring redox homeostasis in the diseased liver.

## 2. Globin Family and Redox Biology

### 2.1. Brief Overview of the Globin Family: Traditional vs. Non-Traditional Roles

**Figure 2:**
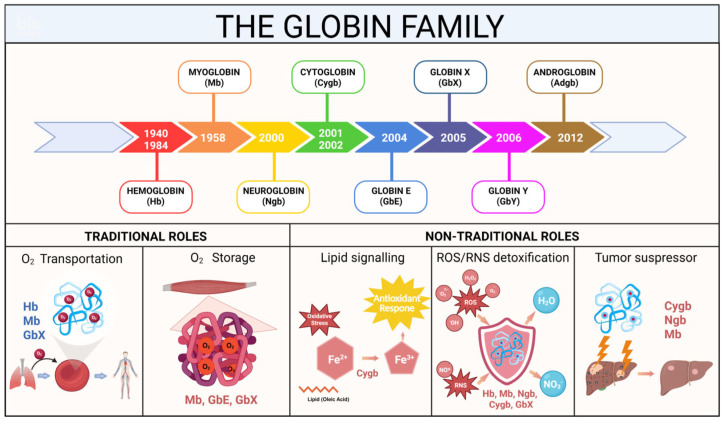
The timeline discovery of the globin family and overview about some traditional and non-traditional functions: Oxygen transportation, Oxygen storage, Lipid signaling; Reactive Oxygen Species (ROS) and Reactive Nitrogen Species (RNS) detoxification, Tumor suppressor. Created in BioRender. Education, G. (2026) https://BioRender.com/t04egtm, accessed on 23 February 2026.

The globin family comprises a group of heme proteins, characterized by a polypeptide chain folded into α-helices surrounding a protoporphyrin IX heme group with a central iron ion (Fe^2+^) [23]. Initially, attention was focused on traditional globins, such as HB in red blood cells and MB in muscle cells, whose primary functions are the transport (HB) and storage (MB) of molecular oxygen (O_2_) in blood and muscle tissue, respectively. In recent years, six other globin types have been identified, including NGB [11], CYGB [6,24,25], Globin X (GBX) [26], Globin Y (GBY) [27], eye-globin or Globin E (GBE) [28], and Androglobin (ADGB) [29]. Along with the discovery of these new globin types, many non-traditional functions have also been uncovered (Figure 2). The expanding knowledge on globin functions suggests that these globins are more than oxygen-binding proteins but instead possess novel functions, including nitric oxide (NO) metabolism, ROS detoxification, peroxidase activity, tumor suppression, apoptosis inhibition, and signaling functions. HB, MB, NGB, CYGB, and GBX can scavenge harmful NO radicals and oxygen species. These proteins, particularly NGB, act as potent anti-apoptotic proteins, particularly when cells are confronted with stressful conditions such as oxidative stress or vascular injury [23]. In addition, by potentially regulating nitric oxide (NO), oxidative stress, or oxygen signaling, the expression of CYGB and MB in cancer cells may function as a tumor-suppressive mechanism by hindering their proliferative capacity [30,31]. Our research in a murine Cygb knockout model also revealed that the absence of CYGB expression resulted in an increased incidence of tumor development in the liver and lungs [32]. Moreover, CYGB may trigger a lipid signaling cascade. While HB, MB, NGB, CYGB, and ADGB are widespread and may be present in most gnathostome (jawed) vertebrates, the GBE, GBX, and GBY types are restricted to lower vertebrate groups, and their functions have yet to be fully elucidated [33].

### 2.2. Structural and Functional Features Relevant to Antioxidant Activity

Among the diverse functions of globins, their antioxidant capacity is currently one of the most focused and frequently studied roles in diseases, particularly for intracellular globins like MB, NGB, and CYGB because of the important associations between the redox activity of the globin family and cell stress response mechanisms [34]. The antioxidant and redox-regulatory functions of the globin family are strictly dictated by their structural characteristics. All globins contain a heme group capable of cycling between Fe^2+^ (reduced) and Fe^3+^ (oxidized) states, providing the foundation for electron-transfer reactions, free radical scavenging, and redox signaling within the intracellular environment [35]. NGB, GBX, ADGB, and CYGB exhibit a hexacoordinate pattern in which the heme iron (both Fe^2+^ or Fe^3+^ oxidation states) is stably anchored by both proximal and distal histidines (histidine or glutamine in E7) [33]. This configuration reduces the ability of Fe^2+^ to bind to exogenous ligands and simultaneously increases its flexibility to participate in redox reactions, thereby facilitating the catalytic degradation of oxidative agents by globins [35,36]. Therefore, hexacoordinated globins, especially NGB and CYGB, participate in antioxidant reactions more strongly than other globins. In addition, CYGB and NGB contain two cysteines (Cys-38 and Cys-83 for CYGB and Cys-46 and Cys-55 for NGB) that can form intramolecular or intermolecular disulfide bridges [37,38]. The formation of these bonds decreases the distal histidine binding affinity to heme iron; therefore, the substitution or reduction in these residues markedly decreases their affinity for O_2_ [39,40,41]. This indicates that the cellular redox state influences protein structure by S-S bond formation or cleavage, ultimately affecting O_2_ binding.

Moreover, under oxidative conditions, oxidation of CYGB to the ferric state facilitates its interaction with lipids and subsequent lipid peroxidation, generating electrophilic oxylipids that can trigger cell signaling pathways and up-regulate an antioxidant response [42]. Owing to these structural features, several globins possess direct and distinct antioxidant capacities through their peroxidase and NO dioxygenase activities, enabling efficient scavenging of a wide range of free radicals, including the [2,2′-azino-di-(3-ethyl-benzthiazoline-6-sulfonic acid)] ABTS^+^ cation, superoxide anion, hydrogen peroxide (H_2_O_2_), and hydroxyl radicals [18,43]. From this, their antioxidant function serves to shield cells and crucial structures such as neurons and the heart from injury, inhibiting apoptosis and sustaining cell viability during stress.

### 2.3. ROS/RNS Detoxification Mechanisms Across the Family

The redox activity of the wider family of globins also has important associations with ROS and RNS scavenging mechanisms. A range of mechanistic strategies has been described for ROS/RNS detoxification. In globin members HB, MB, CYGB, NGB, and GB X, the heme can interact directly with ROS such as hydrogen peroxide and superoxide (O^2−^), leading to partial detoxification; however, this reaction may also generate a hypervalent ferryl heme and protein-based radicals [27,44].

P-Fe^2+^ + H_2_O_2_ ↔ P-[Fe^4+^= O^2−^]^2+^ + H_2_O

P-Fe^3+^ + H_2_O_2_ ↔ P^•+^-[Fe^4+^= O^2−^]^2+^ + H_2_O

In addition, HB and MB can detoxify RNS, such as potentially harmful NO radicals, by converting them into innocuous nitrate (NO_3_) via their dioxygenase activity. HbFe(III)–OO^•^, HbFe(III)–OONO, and protein-caged [HbFe(III)–O^••^NO2] are proposed intermediates in a reaction mechanism that combines both O-atoms of O_2_ with NO to form nitrate and HbFe(III) [45]. Similarly, CYGB and NGB may scavenge RNS or ROS. The intramolecular disulfide bonds formed by cysteine residues in both CYGB and NGB are hypothesized to regulate the binding of exogenous ligands and modulate their respective nitrite reductase or ROS scavenging activities [14]. They also function as NO dioxygenases to scavenge NO under normoxic conditions.

Fe^2+^-O_2_ + NO ⇌ Fe^3+^-ONOO^−^ → Fe^3+^ + NO_3_^−^

Furthermore, under hypoxic conditions, they can generate NO, particularly NGB. When the oxidized disulfide bridge decreases the binding affinity of the distal histidine for the heme iron, NGB adopts the pentacoordinate state, which increases the nitrite reductase activity [46].

Fe^2+^ + NO_2_^−^ + H^+^ → Fe^3+^ + OH^−^ + NO

This helps to maintain NO homeostasis and protect cells from oxidative stress. The exact detoxification mechanisms of ROS and RNS by the newer globins (ADGB, GBY, and GBE) are largely unclear and are currently under investigation.

### 2.4. CYGB and NGB as Redox-Active Globins

The redox activity inherent to globins, particularly the human globins, NGB and CYGB, is highly beneficial for cell survival under stress conditions. Over the last two decades, the expanded globin family has drawn considerable attention to the protective functions of NGB and CYGB in disease states. Redox regulation of CYGB and NGB was first demonstrated through the formation of an intramolecular disulfide bond [37]. NGB is predominantly found in the central and peripheral nervous systems, cerebrospinal fluid, retina, and endocrine tissues [11]. NGB regulates NO and scavenges ROS, providing neuroprotection, restricting cell death caused by oxygen-glucose deprivation and hypoxia, and safeguarding against ischemic injury and neurodegenerative diseases [14,15]. NGB also reduces tissue infarction and mitigates oxidative stress markers following stroke [47]. CYGB acts as a peroxidase, consuming hydrogen peroxide and lipid peroxides, thereby reducing oxidative stress. It may be used as a therapy to prevent liver fibrosis progression because peroxides can activate hepatic stellate cells (HSCs), which promote liver fibrosis [6,48]. CYGB also plays a critical role in the regulation of vascular tone and blood pressure regulation via NO metabolism [49].

## 3. Antioxidant and Cytoprotective Roles of Cytoglobin in the Liver

### 3.1. Antifibrotic and Antitumor Effects of Cytoglobin Observed in Experimental Systems

Since redox reactions activate the extracellular matrix-producing myofibroblasts in liver fibrosis [5], CYGB’s natural regulation of oxidative stress makes it a promising, globally researched therapy. An in vitro model conducted by Ninh Quoc Dat et al. [9] revealed that recombinant human Cytoglobin (rhCYGB) inactivated HSCs and limited fibrosis by scavenging ROS, thereby protecting human hepatic stellate cells (HHSteCs) and mouse hepatic stellate cells (mHSCs) from hypoxanthine-xanthine oxidase and H_2_O_2_-induced cytotoxicity. Mechanistically, His-CYGB was shown to promote interferon-beta (IFN-β) secretion via activation of TANK-binding kinase 1 (TBK1), which subsequently triggered the JAK/STAT signaling pathway in HHSteCs. This signaling cascade contributed synergistically to HSC inactivation and the downregulation of fibrosis-associated gene expression. This study also demonstrated that replacing iron in the heme center of rhCYGB with cobalt abolished this function, confirming that heme is crucial for CYGB function [9]. In another study, under conditions of spontaneous activation, TGF-β1 stimulation, or treatment with tert-butyl hydroperoxide (TBHP), an organic H_2_O_2_ compound, HHSteCs exhibited increased COL1A1 promoter activity accompanied by a time- and dose-dependent rise in COL1A1 protein levels. These effects were significantly attenuated when HHSteCs were cotreated with rhCYGB [10]. The study by Yoshinori Okina et al. demonstrated that overexpression of CYGB in HHSteCs and LX-2 cells transfected with a pCMV6 vector encoding human CYGB significantly attenuated the increase in ROS induced by rhTGF-β1 or H_2_O_2_. Consistently, Western blot analysis of cell lysates obtained under the same experimental conditions showed that rhTGF-β1 or H_2_O_2_ induced upregulation of α-SMA and COL1A expression, whereas CYGB overexpression markedly suppressed this induction at both the protein and mRNA levels [50]. Following our previous studies, in vivo experiments have also demonstrated the antifibrotic effects of CYGB across various models of fibrosis induction. In thioacetamide (TAA)-induced liver fibrosis models, *Cygb* overexpression protected *Cygb* transgenic mice against fibrosis by limiting extracellular matrix production via activation of the nuclear factor erythroid 2 related factor 2 (NRF2) pathway and its target, glutathione peroxidase 2 (Gpx-2), while inhibiting the accumulation of 4-hydroxynonenal (4-HNE) and 8-hydroxy-2′-deoxyguanosine (8OhdG) [8]. Treatment of TAA-induced fibrotic mice with His-CYGB reduced oxidized glutathione (GSSG) and increased the ratio of reduced glutathione (GSH) to GSSG, an important ROS scavenger. The expression levels of lipid peroxidation (4-HNE), collagen deposition (Sirius Red), and alpha-smooth muscle actin (aSMA) were also significantly reduced following His-CYGB treatment [9]. In the choline-deficient, L-amino acid-defined (CDAA) diet mouse model conducted by Le Thi Thanh Thuy et al., CYGB loss promoted SMAD3 activation and enhanced oxidative and hypoxic stress in HSCs, predisposing the liver to progressive fibrosis by increasing expression of fibrogenic genes [48]. In the two mouse models of liver injuries, including 3,5-Diethoxycarbonyl-1,4-dihydrocollidine diet-induced cholestasis [9] and carbon tetrachloride (CCl_4_)-induced fibrosis [10], rhCYGB exerted protective effects by reducing HSC activation, blocking inflammatory responses, and inhibiting fibrosis development. Li et al. also demonstrated that rhCYGB treatment effectively reversed fibrosis in CCl_4_-induced liver fibrosis in a rat model [51]. Using two-dimensional electrophoresis (2-DE) and gene ontology analysis, the stage of hepatic damage and liver fibrosis in the rhCYGB group was found to be significantly milder than that in the CCl_4_ model group [51]. In a bile duct ligation–induced fibrosis model, Van Thuy et al. found that *Cygb* deficiency enhances liver injury and fibrogenesis during cholestasis in mice via the deleterious effects of NO, bile canalicular function impairment, and excessive toxic bile acid accumulation in hepatocytes [52]. Interestingly, aged *Cygb*-deficient mice exhibited widespread organ damage, including spontaneous liver fibrosis [32]. Serum and urine analysis in these *Cygb*-deficient mice demonstrated that the concentration of NO metabolites increased significantly compared to their wild-type counterparts, resulting in an imbalance in the oxidative stress and antioxidant defense system that was reversed by NG-monomethyl-L-arginine treatment. A senescent phenotype and evidence of DNA damage have been found in primary HSCs and the liver of aged Cygb-deficient mice [32]. Collectively, these in vitro and in vivo findings demonstrate that CYGB exerts a robust antifibrotic effect across multiple experimental models of liver injury. By maintaining redox homeostasis through potent ROS and NO scavenging, activating antioxidant pathways such as NRF2, and suppressing HSC activation and fibrogenic signaling pathways, CYGB has emerged as a potential therapeutic target for chronic liver disease and a key regulator of liver fibrosis progression.

Although basal ROS levels are required for normal cellular signaling, excessive ROS accumulation induces oxidative stress, DNA damage, lipid peroxidation, and chronic inflammation, thereby promoting malignant transformation and tumor progression [53,54]. CYGB, through its role in regulating ROS, functions as a tumor suppressor across multiple experimental models. Zhang et al. investigated the role of CYGB in HCC cell lines such as HepG2, HuH7, and PLC/PRF/5. The results showed that CYGB suppressed cell proliferation and reduced sphere-forming capacity, whereas CYGB downregulation promoted cell proliferation. CYGB also inhibited the expression of key stemness markers, including OCT4, SOX2, and β-catenin, and simultaneously suppressed the activation of PI3K/AKT signaling. Notably, these inhibitory effects were abolished upon GSH supplementation, indicating that the tumor-suppressive effects of CYGB are mediated through the regulation of ROS/RNS-dependent oxidative–nitrosative stress pathways [55]. Another study conducted in 2025 on HCC cell lines, including HepG2, Huh7, and Hep3B, further confirmed the tumor-suppressive role of CYGB via its antioxidant mechanisms. This study also demonstrated that CYGB inhibits HCC by reducing lipid peroxidation, suppressing cell proliferation, and inactivating the AKT/ERK/Cyclin D1 axis, thereby controlling proliferative signaling [56]. The tumor-suppressive role of CYGB has been demonstrated in vivo across multiple models of liver cancer induced by diverse agents. In the CDAA diet mouse model, *Cygb*-deficient mice progressed rapidly from steatohepatitis to liver cancer and exhibited early DNA double-strand damage markers (53BP1, γ-H2AX). Disturbances in ERK and AKT signaling, along with elevated expression of cytokines such as IL-1β, IL-6, TNFα, and TGFβ3, contributed to a tumor-promoting environment [48]. In addition, altered expression of cell–cycle–related genes, including *p53*, *cyclinD2*, *Pak1* (p21-activated kinase), *Src*, *Cdkn2a*, and *Cebpa* (CCAAT/enhancer-binding protein α), together with enhanced nitrosative stress, is considered a major mechanism driving liver tumorigenesis in *Cygb*-deficient mice [7]. These results suggest a novel and promising strategy for cancer treatment, in line with the development of recombinant protein-based therapies [7].

### 3.2. CYGB Expression Inversely Correlates with Disease Severity in Humans

CYGB is central to cellular responses to stress and tissue injury. Consequently, analyzing the relationship between CYGB expression and disease severity in human cohorts provides critical insights into the role of CYGB in disease progression and its potential as a clinical biomarker.

MASH is a progressive liver injury that serves as a precursor to cirrhosis and hepatocellular carcinoma (HCC) [57]. During this progression, the generation of ROS and subsequent oxidative stress, along with multiple signaling pathways, play a crucial role in activating hepatic stellate cells (HSCs)—the primary drivers of excessive extracellular matrix deposition and tissue remodeling [58]. Given its function as a ROS scavenger, the role of CYGB in MASH has been extensively studied. Le Thi Thanh Thuy et al. used immunohistochemistry to analyze liver samples from 15 patients with MASH and found that CYGB expression significantly decreased as MASH scores increased. A parallel reduction in CYGB mRNA and protein levels was observed in HCC regions, suggesting that CYGB loss may facilitate the development and progression of both MASH and HCC [48]. Okina et al. demonstrated that markers of lipid peroxidation (4-HNE) and oxidative DNA damage (8-OHdG) rose in tandem with histological fibrosis (Metavir scores) in a cohort of patients with NAFLD/MASH. The downregulation of CYGB in these patients was attributed to the TGF-β1/SMAD2/SP3-M1 signaling pathway, which led to the accumulation of hydroxyl radicals (•OH) and DNA damage in HSCs [50]. Research by Motoyama et al. involving liver biopsies from 40 patients with HCV identified CYGB as a reliable marker for quiescent human HSCs. The density of CYGB-positive cells was inversely correlated with fibrosis progression, with a marked decline from Metavir stages F1 to F4 [59]. In a study of 123 patients with HCC, Yang et al. reported that CYGB levels were significantly lower in tumor tissues than in adjacent non-tumor regions. Reduced CYGB expression was associated with aggressive characteristics, including larger tumor size, advanced clinical stage, and poor differentiation. Furthermore, bioinformatic analysis revealed that patients with low CYGB expression had significantly worse overall survival [56].

The tumor-suppressive capacity of CYGB extends beyond the liver, as demonstrated in various in vitro and in vivo models (Table 1). Hoang et al. found that CYGB-overexpressing transgenic mice had a significantly lower incidence of DMBA-induced pancreatic tumors than wild-type mice, likely due to the ability of CYGB to inhibit inflammation and reduce oxidative DNA damage [60]. In melanoma cell lines, Zou et al. demonstrated that CYGB suppresses proliferation and invasion by regulating the ZEB1/GPX4 axis and inhibiting the epithelial–mesenchymal transition. Interestingly, CYGB also conferred ferroptosis resistance in these cells by reducing the labile iron pool and levels of ROS [61]. Fan et al. reported that CYGB promotes ferritinophagy and interacts with the transferrin receptor to facilitate ferroptosis-mediated cell death in colorectal cancer lines [62]. In breast cancer models, CYGB is often silenced by promoter methylation; its restoration inhibits tumor growth and induces apoptosis by suppressing glucose metabolism (via GLUT1/HK2 downregulation) through p53-dependent pathways [63]. Similarly, in lung cancer, CYGB gene silencing via methylation is associated with increased colony formation, while its enforced expression serves a protective, tumor-suppressive function [64]. Collectively, these findings highlight CYGB as a versatile protective protein. By modulating oxidative stress, inflammation, autophagy, and metabolic pathways, CYGB functions as a broad-spectrum tumor suppressor and a promising therapeutic target for various malignancies.

## 4. Antioxidant and Cytoprotective Roles of Neuroglobin in the Liver

Discovered almost concurrently with CYGB, NGB, another member of the globin family predominantly expressed in the brain, plays a crucial role in protecting brain cells from ischemic and oxidative stress [11,67]. As discussed in the previous section regarding the role of ROS in liver diseases and the function of CYGB as a ROS scavenger, NGB also exhibits this capability due to its structural similarity to CYGB, specifically its hexacoordinate heme iron pattern. Although its function in the nervous system is well-documented, its potential therapeutic application in hepatic pathologies remains a novel area of investigation.

### 4.1. Expression and Antioxidant Effects of Neuroglobin

NGB is a widely distributed protein found not only in the central and peripheral nervous systems [11,68] but also in various normal body tissues and several cancer cell lines [12]. Functionally, NGB serves as a primary physiological sensor for hypoxia and oxidative stress, mediating cellular responses via conformational transitions and subsequent interactions with signal transduction pathways, notably G-proteins [13]. Upon exposure to severe hypoxic or oxidative insults, NGB expression is significantly upregulated, promoting its mitochondrial translocation where it binds cytochrome c to competitively inhibit apoptotic cascades [16,17]. Furthermore, NGB demonstrates nitrite reductase activity, catalyzing the generation of nitric oxide (NO) to modulate and attenuate cellular respiration [14]. Concurrently, NGB acts as a robust endogenous antioxidant, facilitating direct free radical scavenging and Fe^2+^ chelation. These multifaceted cytoprotective mechanisms underscore the intrinsic neuroprotective efficacy of NGB [18]. Consequently, targeted delivery or upregulation of NGB is being actively explored as a novel therapeutic strategy to combat various pathologies, including neurological conditions like ischemic stroke and Alzheimer’s disease, cardiovascular disorders like myocardial ischemia and cardiac hypertrophy, and hepatic diseases such as HCC and liver fibrosis.

NGB upregulation not only confers cytoprotection but also fundamentally alters intracellular signaling and metabolic processes. A 2006 in vitro study by Bin Cai et al. identified NGB as a metabolic regulator in mouse hippocampal neurons. This mechanism inhibits AMPK signaling and activates acetyl-CoA carboxylase, thereby promoting anabolic processes, specifically lipid and glycogen accumulation, to enhance cellular energy reserves [69]. Furthermore, NGB functions as a tumor suppressor in specific malignancies by modulating stress pathways. For instance, a study published in Biochem Biophys Res Commun (2023) reported that NGB enhances sensitivity to epidermal growth factor receptor (EGFR) inhibitors in pancreatic cancer cell lines by targeting the GNAI1/EGFR/AKT/ERK axis [70]. Similarly, Li et al. reported that NGB overexpression inhibits cell growth and invasion by promoting autophagy via inactivation of the EGFR/PI3K/AKT/mTOR signaling pathway in GBM cell lines. Conversely, Ngb knockdown significantly increased cell proliferation rates, accelerated cell cycle entry into the S phase, and enhanced tumor invasiveness [71]. Sun et al. (2001) further corroborated this protective function; they observed a drastic increase in hypoxia-induced cell death accompanied by robust caspase-3 activation using antisense oligonucleotides to deplete NGB in cortical neurons [67].

A study by Fordel et al. (2007) using Swiss-CD1 mice elucidated the relationship between NGB and hepatic oxidative stress under conditions of chronic hypoxia. Although NGB exhibited a slight upregulation in response to hypoxia, its mRNA expression levels progressively declined during the reoxygenation phase. The hepatic production of ROS is significantly higher than that in other organs, such as the brain, eyes, or heart. Given that the liver is subjected to substantial oxidative stress, the inability to sustain elevated NGB levels during reoxygenation results in antioxidant system being overwhelmed, which leads to hepatocellular injury and the activation of programmed cell death (apoptosis), as confirmed by increased caspase-3 activity [72].

Okogwu et al. (2014) used a goldfish (*Carassius auratus*) model to investigate the impact of toxin exposure combined with hypoxia. Following injection with the cyanobacterial toxin Microcystin-RR (MC-RR) under hypoxic conditions, Ngb mRNA was robustly upregulated only in the brain—increasing several-fold to protect the nervous system from oxidative damage. In contrast, NGB levels in the liver and other organs remained unchanged or did not increase sufficiently. The combination of MC-RR toxin and hypoxia induced a synergistic effect in the goldfish liver. Consequently, the lack of an NGB upregulation response renders the liver “unprotected” and more susceptible to necrosis and apoptosis than the brain, where NGB functions as an effective protective shield [73].

Follow our previous studies in Redox Biology [10] demonstrates that the administration of rhNGB exerts antioxidant effects, thereby attenuating the liver fibrosis process and is effective both in vitro and in vivo. Regarding in vitro evidence in HSCs, the cell type primarily responsible for liver fibrosis, rhNGB reveals its remarkable capacity for cellular internalization and its protective effect against oxidative stress. rhNGB is capable of entering HSCs and localizing to the membrane, cytoplasm, and nucleus. These biochemical properties translate into effective cytoprotection. HHSteCs generate ROS spontaneously during culture; rhNGB treatment effectively suppressed this basal ROS accumulation. To simulate acute oxidative stress, cells were challenged with TBHP, an organic peroxide, or stimulated with the pro-fibrotic cytokine TGF-β1. Assays utilizing the fluorescent probes DCFDA (for total ROS) and Bes-H_2_O_2_ (specifically for hydrogen peroxide) revealed that NGB pre-treatment significantly blunted the intracellular surge of ROS induced by these stressors [10]. Having established the cellular mechanisms in vitro, in vivo experiment evaluated the behavior of rhNGB in a living system. To assess therapeutic potential, an in vivo chronic liver fibrosis model was established in C57BL/6 mice using CCl4 for six weeks, and rhNGB treatment demonstrated significant therapeutic efficacy. Mechanistically, NGB treatment lowered the levels of lipid peroxidation markers (4-HNE), confirming its in vivo antioxidant role [10].

In the pathology of HCC, ROS are well-recognized as the primary drivers of disease promotion and progression. Zhang et al. demonstrated that the antioxidant function of NGB extends beyond simple free radical scavenging; crucially, NGB acts as a sensor for oxidative stress to regulate intracellular signaling. In vitro experiments using H_2_O_2_ (a specific ROS) to stimulate hepatic cells, it was observed that H_2_O_2_ typically triggers robust activation of the Raf/MEK/ERK pathway—a critical cascade for tumor development. However, NGB overexpression abolished this H_2_O_2_-induced ERK activation. This finding provides evidence that NGB interposes between oxidative stress signals and the cellular proliferation machinery. Unlike conventional antioxidant enzymes, such as catalase or superoxide dismutase, which primarily eliminate ROS, NGB functions as a molecular “switch.” It senses intracellular ROS and oxygen levels. NGB undergoes a conformational change upon binding to oxygen or ROS that enables physical interaction with c-Raf—a key initiator of oncogenic pathways. This interaction effectively sequesters c-Raf, thereby preventing downstream signal transduction [74].

### 4.2. Human NGB Suppresses Liver Fibrosis and Cancer

In terms of how rhNGB exerts its anti-fibrotic effects, the reduction in fibrosis observed in vivo is driven by distinct molecular mechanisms governing collagen dynamics. By scavenging intracellular ROS, rhNGB inhibits COL1A1 gene promoter activity, leading to reduced COL1A1 expression at both the mRNA and protein levels. Mutations disrupting the disulfide bond of NGB reduce ROS scavenging capacity and simultaneously abolish the ability to inhibit collagen, confirming the close link between antioxidant and antifibrotic properties. As a key novel finding, rhNGB stimulated HSCs to secrete matrix metalloproteinase-1 (MMP-1), a crucial enzyme in collagen degradation. Inhibition of MMP-1 using siRNA attenuated the antifibrotic efficacy of NGB, demonstrating that NGB operates via a dual mechanism: reducing collagen synthesis while enhancing its degradation. Transcriptomic analysis further revealed that rhNGB induces deactivation of HSCs by downregulating fibrosis-related genes (e.g., COL1A2, TGF-β3, and PDGFRα) and upregulating genes associated with HSC deactivation, such as PPARg and ETS1. Additionally, similar to CYGB, NGB activated the interferon a/b signaling pathway. Histological analysis using Sirius Red staining revealed a marked reduction in collagen deposition and bridging fibrosis in NGB-treated mice compared with controls. This was quantitatively confirmed by a significant decrease in hepatic hydroxyproline levels. Furthermore, rhNGB suppressed HSC activation, as evidenced by reduced aSMA expression, and attenuated inflammatory cell infiltration [10].

In the context of HCC, Zhang et al. demonstrated that NGB functions as a tumor suppressor by binding to c-Raf-1, thereby inhibiting activation of the Raf/MEK/ERK signaling pathway, which is a known driver of cell proliferation. In vitro experiments revealed that silencing Ngb expression in HepG2 cells (an HCC cell line) using shRNA resulted in enhanced cell growth and proliferation, while simultaneously accelerating the transition of the cell cycle from the G0/G1 phase to the S phase. Conversely, NGB overexpression achieved via plasmid transfection inhibited HepG2 cell growth and proliferation and significantly reduced colony formation capabilities in soft agar assays. These findings were corroborated in vivo using a xenograft model in female BALB/c nude mice aged 4–5 weeks. Following the subcutaneous injection of HepG2 cells with modulated Ngb expression, the results mirrored the in vitro data, in that the knockdown group exhibited accelerated growth compared with the control group. Tumorigenicity was significantly suppressed in the overexpression group, with the tumor size and weight being markedly lower than those of the control group. Collectively, these results confirm the potential of NGB as a suppressor of HCC [74].

In conclusion, studies on NGB in humans have primarily focused on neurological disorders. Research on the application and impact of NGB on hepatic pathologies remains limited, highlighting this as a highly promising avenue for future investigation (Table 2). The utility of rhNGB for treating liver fibrosis, particularly HCC, suggests that it is a promising new therapeutic target for liver diseases.

## 5. Therapeutic Implications and Applications

Oxidative stress is a key pathogenic driver of chronic liver diseases, contributing to fibrosis progression and hepatocarcinogenesis [9,10,50]. Although conventional antioxidants have been extensively investigated, their clinical benefits remain limited, underscoring the need for more effective strategies that regulate oxidative stress responses rather than passively scavenging free radicals [80].

**Figure 3:**
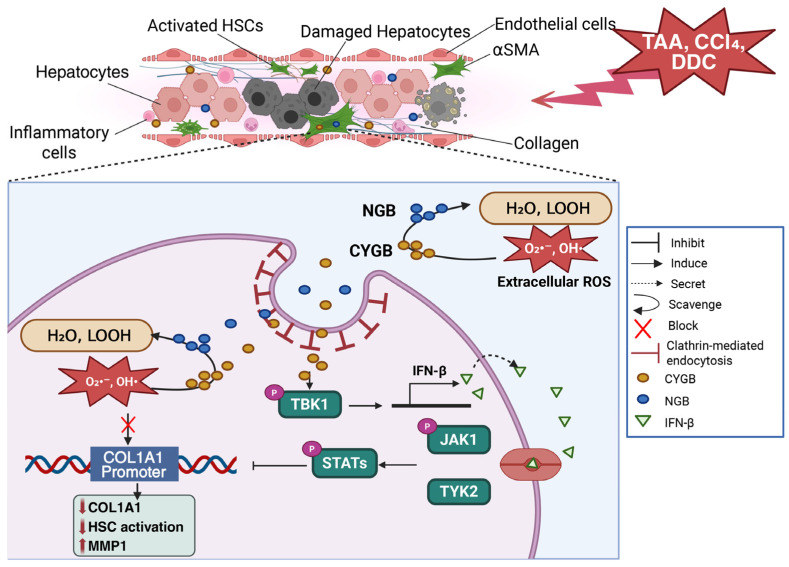
CYGB and NGB scavenged ROS, deactivated HSCs, and inhibited liver fibrosis. NGB and CYGB were endocytosed into the HSCs via endocytosis pathway, scavenged extracellular and intracellular ROS, and suppressed *COL1A1* promoter activity, resulting in HSC deactivation and inhibition of collagen production. MB and NGB also promoted the upregulation of matrix metalloproteinase 1 (MMP1) gene involved in collagen degradation in the extracellular matrix. Additionally, CYGB induced Interferon-beta (IFN-β) secretion mediated by TANK binding kinase 1 (TBK1), which subsequently triggered the JAK/STAT signaling pathway resulting in HSC deactivation and inhibition of collagen gene expressions. Created in BioRender. Education, G. (2026) https://BioRender.com/xh6zo9q, accessed on 27 February 2026.

In this context, intracellular redox-active globins have gained increasing attention as potential therapeutic approaches due to their ability to modulate redox-sensitive signaling pathways. CYGB and NGB represent stress-responsive globins within this broader class. Experimental evidence summarized above demonstrates that these globins regulate redox homeostasis, suppress HSC activation, and attenuate fibrogenic and proliferative responses in liver disease models. Their anti-fibrotic efficacy, demonstrated across CCl_4_, TAA, BDL, and CDAA models representing chemotoxic, cholestatic, and metabolic injury, suggests broad translational relevance across etiologically distinct human liver diseases (Figure 3, [9,10]).

### 5.1. Recombinant Protein-Based Therapies: Barriers and Feasibility

Recombinant protein therapies provide highly specific and potent interventions in disease pathways [81]. The classic example is insulin, a life-saving hormone therapy that heralded the recombinant biologics era [82]. To date, comprehensive databases have documented 894 FDA-approved therapeutic proteins as of 2024, including 354 monoclonal antibodies and 85 peptides or polypeptides, reflecting the substantial growth and maturation of this therapeutic class over the past two decades [83]. Unlike small molecules, protein drugs (especially enzymes) can catalyze biochemical reactions, but delivering them into cells remains challenging. Proteins are typically too large and polar to cross cell membranes and are susceptible to proteolysis and rapid clearance [81]. In particular, unmodified recombinant proteins with molecular masses below 50 kDa, a category that includes both rhCYGB (~21.5 kDa) and rhNGB (~17 kDa), typically exhibit terminal serum half-lives in the range of minutes to hours in vivo, driven primarily by renal filtration and proteolytic degradation [9,10]. Thus, most approved biologics act on extracellular targets, often requiring parenteral administration [84]. Additionally, immunogenicity and manufacturing complexity pose major hurdles [81,82], while advanced delivery and engineering strategies have emerged to overcome these barriers. Enzyme replacement therapies exemplify intracellular targeting by exploiting receptor-mediated uptake (mannose-6-phosphate tags) to deliver enzymes into lysosomes [85]. Many recombinant proteins are chemically modified, for example, PEGylation, the first successful half-life extension technology with over 25 years of clinical application, can reduce renal filtration by increasing hydrodynamic radius while shielding proteins from protease access [81]. Similarly, fusion to immunoglobulin Fc domains or human serum albumin (HSA) exploits the neonatal Fc receptor (FcRn) recycling pathway, extending the therapeutic half-life to approximately 21 days, comparable to the duration of endogenous IgG and albumin [86]. Such strategies have produced clinically validated improvements: pegfilgrastim (PEGylated G-CSF) replaced its unmodified predecessor Neupogen^®^ by enabling once-per-cycle rather than daily dosing, while Fc-fusion cytokines such as etanercept demonstrated that intracellular signaling targets can be successfully addressed by engineered extracellular delivery. These globins act within cells to scavenge ROS, showing promise for mitigating oxidative stress–driven liver damage [9,10]. However, despite this cell-penetrating capacity, the overall delivery efficiency to the fibrotic hepatic niche remains stoichiometrically suboptimal when administered as naked proteins systemically, as the dense ECM of advanced fibrosis impairs macromolecular distribution [81]. Similarly to other recombinant proteins, CYGB and NGB face similar delivery and stability barriers, but their catalytic redox activity highlights the innovative potential of next-generation protein therapeutics [9,10].

Notably, the high intrinsic thermal stability of NGB (melting temperature Tm ≈ 100 °C) and CYGB (Tm ≈ 95 °C), values far exceeding those of most recombinant therapeutic proteins, confers a significant advantage in terms of manufacturing robustness and formulation stability [87]. To surmount pharmacokinetic hurdles, the integration of advanced bioengineering and nanomedicine is paramount for future clinical success. Promising platforms under preclinical investigation include lipid nanoparticle (LNP) encapsulation for hepatic tropism; AAV8-mediated gene delivery for efficient liver transduction; glycoengineering to enhance targeted cellular uptake; and structural modifications such as PEGylation or HSA/Fc-fusion engineering to extend circulating half-life while preserving redox function [86,88,89].

### 5.2. Pathway to Clinical Translation and Projected Timeline

Building on the therapeutic potential and translational challenges of recombinant protein-based therapies discussed above, successful clinical development ultimately depends on a well-defined and regulator-guided translational pathway.

The clinical development of CYGB- and NGB-based therapies will follow a stepwise translational pathway common to modern biologics, encompassing delivery system optimization, scalable GMP manufacturing, and rigorous safety evaluation. Early clinical exploration would likely focus on patients with advanced but compensated disease, where oxidative stress plays a dominant pathogenic role and therapeutic windows remain accessible [90].

Recent regulatory approvals highlight the feasibility of translating mechanistically targeted biologics into liver medicine. The FDA approval of TSHR-targeting antibodies and GLP-1 receptor agonists highlights the successful transition of protein- and peptide-based therapies from pathophysiological insights to clinical practice, even in complex metabolic and endocrine contexts. These precedents suggest that globin-based redox modulators, once supported by appropriate pharmacokinetic and biomarker frameworks, could follow a similar trajectory [91].

Looking forward, CYGB and NGB represent promising candidates for the development of first-in-class redox-modulating biologics in hepatology. Their ability to directly restore intracellular redox balance, protect multiple hepatic cell types, and synergize with existing therapies position them as realistic drug candidates rather than purely experimental antioxidants. The continued integration of advances in bioengineering, targeted delivery strategies, and translational biomarkers will ultimately determine whether globin-based therapies can be successfully advanced into early-phase clinical trials and, eventually, clinical practice [9,10].

## 6. Challenges and Future Directions

Despite promising experimental evidence supporting the use of CYGB and NGB in liver pathologies, several hurdles remain for their clinical translation. First, there is still a limited understanding of how globin expression is regulated in different liver disease states. Specifically, while the neuroprotective role of NGB is well documented, data on its function and regulation in hepatic tissue require further investigation. Second, CYGB has shown “bimodal” behavior; it is often downregulated in normoxia as a tumor suppressor but can be elevated in hypoxia, potentially promoting tumor progression in certain contexts. Clarifying these conditions is vital for safe therapeutic application. Third, as these globins are expressed in multiple organs (NGB in the brain/retina; CYGB in the heart/brain), the systemic safety of delivering recombinant versions or using gene therapy must be rigorously evaluated to avoid cross-organ side effects. Finally, overcoming the pharmacokinetic barriers of recombinant proteins—such as membrane permeability and short half-lives—requires advanced bioengineering. To ensure site-specific delivery, future research should focus on synthetic globin mimetics, fusion proteins, or liver-targeted viral vectors such as AAV8.

## 7. Conclusions

In conclusion, CYGB and NGB represent a significant shift in our understanding of the globin family, moving beyond simple oxygen carriers to sophisticated catalytic redox regulators. Their unique hexacoordinate structure and ability to scavenge a wide array of ROS/RNS position them as promising novel therapeutic targets for chronic liver diseases, where traditional antioxidants have failed.

Our research, along with growing global evidence, demonstrates that both CYGB and NGB can effectively deactivate HSCs, reduce collagen deposition, and suppress tumor growth in experimental models. Although challenges in delivery and regulation persist, the integration of these globins into recombinant therapies or gene-based approaches offers a pathway toward restoring redox homeostasis in the diseased liver. Further translational and clinical research is strongly encouraged to realize the potential of these “atypical” globins to improve patient outcomes for fibrosis and HCC.

## Figures and Tables

**Table 1:** Roles of Cytoglobin in vitro and in vivo in recent studies.

Reference	Species	Cells/Organs	Key Findings and Mechanistic Insights
Fan, C. et al., 2025 [62]	Human	Human colorectal cancer cell lines: HCT116 and SW620	CYGB interacts with the transferrin receptor (TFR), thereby activating lysosomal signaling pathways and promoting ferroptosis via the autophagic degradation of ferritin.
Jourd’heuil, F. et al., 2025 [65]	Mouse	Cygb-deficient carotid arteries	CYGB scavenges intracellular hydrogen peroxide, promotes glycolysis and regulates re-dox signals in the vasculature.
Yang, S. et al., 2025 [56]	Human	Normal, HCC tissues Human HCC cell lines: HepG2, Hep3B and Huh7	CYGB inhibits HCC proliferation by suppressing DAMP-induced activation of the oxidative stress–associated AKT/ERK/Cyclin D1 signaling axis. CYGB deficiency correlates with advanced stage, poor differentiation, and poor prognosis.
De Backer, J. et al., 2024 [66]	Human	CYGB-overexpressing melanoma cell line (A375)	CYGB protects against oxidative stress and regulates key inflammasome-associated genes (NLRP1, CASP1, and CD74), as well as cancer-related pathways, including mTORC1 and AKT/mTOR signaling and CSPG4-mediated epithelial–mesenchymal transition (EMT).
Zou, Z. et al., 2024 [61]	Human	Human melanoma cell lines: 16F10, A375 and G361	CYGB suppresses melanoma cell proliferation and inhibits epithelial–mesenchymal transition (EMT), induces ferroptosis resistance by reducing the labile iron pool (LIP) and ROS levels, while upregulating glutathione peroxidase 4 (GPX4) expression.
Hieu, V.N. et al., 2022 [10]	Human	Primary human HSC cell lines	CYGB attenuates intracellular ROS levels through suppression of COL1A1 promoter activity, leading to reduced HSC activation, hepatic inflammation, and fibrogenesis.
	Mouse	CCl_4_-injured livers rhCYGB-treated livers	
Hoang, D.V. et al., 2022 [60]	Human	PDAC tissues, Pancreatic cancer cell lines: BxPC3, MIA PaCa2, PANC1, OCUP-A2 and HpaSteC cell line	CYGB expression in pancreatic ductal adenocarcinoma is negatively correlated with tumor stage and attenuates DMBA-induced tumorigenesis by suppressing inflammatory and fibrotic responses, oxidative DNA damage, and oncogene expression.
Dat, N.Q. et al., 2021 [9]	Mouse	Cygb-deficient livers in BDL/CDAA model Primary mouse HSC cell lines	CYGB scavenges ROS and promotes interferon-beta (IFN-β) secretion via TBK1-mediated activation of the JAK/STAT pathway in HHSteCs, leading to HSC inactivation and downregulation of fibrosis-associated genes.
	Human	Primary human HSC cell lines	
Okina, Y. et al., 2020 [50]	Human	MASH tissues Primary human HSC cell lines	CYGB scavenges hydroxyl radicals (•OH) and reduces 8-OHdG levels, thereby preventing DNA damage, whereas downregulation of CYGB via the TGF-β1/pSMAD2/SP3-M1 pathway induces •OH-dependent oxidative DNA damage in activated hepatic stellate cells of patients with NASH.
Zhang, J. et al., 2019 [55]	Human	HCC, hepatolithiasis tissues Liver cancer stem-like cells, Human HCC cell lines	CYGB suppresses tumor progression through multiple pathways, including PI3K/AKT, SOX2, and β-catenin signaling, in a ROS/RNS-dependent manner.
Thi Thanh Hai, N. et al., 2018 [8]	Mouse	TAA-induced fibrosis model Cygb transgenic livers	CYGB overexpression mitigates HSCs activation and protects against TAA-induced liver fibrosis, likely by maintaining HSCs in a quiescent state.
Feng, Y. et al., 2018 [63]	Mouse	Xenograft tumor model in nude mice (with MB231 cell line)	CYGB expression is significantly reduced in human breast tumor samples, and CYGB suppresses breast cancer progression by inhibiting glucose metabolism.
	Human	Human breast cancer cell lines (MB231, MCF7)	
Van Thuy, T.T. et al., 2017 [52]	Mouse	Liver tissue in BDL model	CYGB deficiency exacerbates liver injury and fibrosis in cholestatic mice by promoting the detrimental effects of nitric oxide, impairing bile duct function, and causing excessive accumulation of toxic bile acids in hepatocytes.
Li, Z. et al., 2016 [51]	Rat	CCl_4_-injured, rhCYGB treated livers	rhCYGB lowers serum ALT, AST, HA, LN, Col III, and Col IV levels and reverses fibrosis during chronic hepatitis through mechanisms involving proteins linked to oxidative stress.
Thuy, L.T.T. et al., 2016 [32]	Mouse	Cygb-deficient aged livers	CYGB maintains redox homeostasis and suppresses tumor development, while its loss can cause organ dysfunction, likely through disruption of nitric oxide and antioxidant systems and induction of accelerated cellular senescence.
Thuy, L.T.T. et al., 2015 [48]	Mouse	CDAA treated Cygb-deficient livers	CYGB expression is inversely correlated with NASH scores in patients. CYGB deficiency induces early DNA damage and activates ERK/AKT signaling pathways, accelerating the progression of fatty liver disease to HCC.
	Human	MASH, HCC tissues	
Motoyama, H. et al., 2014 [59]	Human	HCV and MASH liver tissues LX-2, Huh7 cell lines	CYGB as marker for quiescent stellate cells.
	Mouse	Cygb-overexpressing livers Primary mouse HSC cell lines	
Shivapurkar, N. et al., 2008 [64]	Human	Bronchial and mammary epithelial cell lines	CYGB reduces colony formation in tumor cell lines, indicating its tumor-suppressive role.

PDAC: Pancreatic ductal adenocarcinoma, BDL: Bile duct ligation, CDAA: Choline-deficient, amino acid-defined diet, HCV: Hepatitis C virus.

**Table 2:** Summary of Recent In Vitro and In Vivo Studies Investigating the Roles of Neuroglobin (NGB).

Reference	Species	Cells/Organs	Key Findings and Mechanistic Insights
Li, L. et al., 2025 [71]	Human	Brain/Glioblastoma multiforme (GBM) cells	Proves that NGB overexpression significantly promotes cytoprotective autophagic pathways, subsequently inhibiting tumor cell proliferation, migration, and invasive capacity through the targeted inactivation of the PI3K/AKT/mTOR signaling cascade.
Liang, X. et al., 2024 [75]	Human (In vitro)	Neuronal cells/Mitochondria	Uncovers a profound neuroprotective mechanism where NGB maintains mitochondrial respiratory chain integrity by directly interacting with the mitochondrial complex I-subunit NDUFA10, preventing apoptosis.
Wu, F. et al., 2023 [70]	Human	Pancreas/PDAC cells	Establishes NGB as an extraneural tumor suppressor. Overexpression of NGB enhances cellular sensitivity to EGFR inhibitors by specifically targeting and dismantling the GNAI1/EGFR/AKT/ERK signaling axis.
Gao, W. et al., 2023 [76]	Murine	Brain/HT22 Hippocampal cells	Provides the first evidence that NGB protects cells from ferroptosis. NGB depletion exacerbates erastin toxicity, depletes NRF2 expression, and spikes free iron and malondialdehyde (MDA) levels. Concludes NGB modulates ferroptosis via the NRF2 axis.
Xiang, Q. et al., 2023 [77]	Human	Colon/Colorectal Cancer cells (HCT116, Caco2)	Identifies NGB as an extraneural tumor suppressor that destabilizes GPR35. NGB promotes GPR35 degradation, thereby inhibiting angiogenesis and metastasis.
Hieu, V.N. et al., 2022 [10]	Human and Murine	Liver/Hepatic Stellate Cells (HSCs)	Demonstrates that recombinant NGB internalizes into HSCs, scavenges ROS, completely suppresses COL1A1 promoter activity, and uniquely stimulates MMP-1 secretion for active matrix degradation.
Kim, Y. et al., 2022 [78]	Murine	Brain/Microvascular pericytes	Reveals that the functional distribution of NGB within microvascular pericytes is critically associated with the prevention of blood–brain barrier (BBB) leakage and vascular breakdown following ischemic stroke.
De Simone, G. et al., 2021 [79]	Biochemical	Protein structure	Provides evidence of the “pseudo-enzymatic” properties of NGB, indicating that its ROS-scavenging capabilities are strictly dependent on the heme group’s structural dynamics, forming a defensive barrier for cellular membranes.
Zhang, J. et al., 2013 [74]	Human and Murine	Liver/HepG2 cells, HCC xenografts	Provides seminal evidence that NGB functions as a potent tumor suppressor in the liver. Acts as an oxidative redox sensor that physically binds to c-Raf, thereby intercepting and shutting down the oncogenic Raf/MEK/ERK proliferation pathway.

## Data Availability

No new data were created or analyzed in this study. Data sharing is not applicable to this article.

## Abbreviations

The following abbreviations are used in this manuscript:

2-DE	Two-dimensional electrophoresis
4-HNE	4-Hydroxynonenal
8-OHdG	8-Hydroxy-2′-deoxyguanosine
AAV8	Adeno-associated virus serotype 8
Adgb	Androglobin
AKT	Protein kinase B
ALD	Alcoholic liver disease
AMPK	AMP-activated protein kinase
aSMA	Alpha-smooth muscle actin
BDL	Bile duct ligation
CCl_4_	Carbon tetrachloride
CDAA	Choline-deficient, L-amino acid–defined diet
COL1A1	Collagen type I alpha 1 chain
COL1A2	Collagen type I alpha 2 chain
CYGB	Cytoglobin
DAMPs	Damage-associated molecular patterns
DMBA	7,12-Dimethylbenz[a]anthracene
ECM	Extracellular matrix
EGFR	Epidermal growth factor receptor
EMT	Epithelial–mesenchymal transition
ERK	Extracellular signal–regulated kinase
FDA	U.S. Food and Drug Administration
GbE	Globin E
GbX	Globin X
GbY	Globin Y
GMP	Good Manufacturing Practice
GPx	Glutathione peroxidase
GSH	Reduced glutathione
GSSG	Oxidized glutathione
Hb	Hemoglobin
HCC	Hepatocellular carcinoma
HCV	Hepatitis C virus
HHSteCs	Human hepatic stellate cells
HIF-1α	Hypoxia-inducible factor 1-alpha
His-CYGB	6×His-tagged recombinant cytoglobin
HSA	Human serum albumin
HSCs	Hepatic stellate cells
IFN-β	Interferon-beta
JAK	Janus kinase
LNP	Lipid nanoparticle
MAPK	Mitogen-activated protein kinase
MASH	Metabolic Dysfunction-Associated Steatohepatitis
Mb	Myoglobin
mHSCs	Mouse hepatic stellate cells
MMP-1	Matrix metalloproteinase-1
mTOR	Mammalian target of rapamycin
NAFLD	Nonalcoholic fatty liver disease
NGB	Neuroglobin
NO	Nitric oxide
NOX4	NADPH oxidase 4
NRF2	Nuclear factor erythroid 2–related factor 2
PDAC	Pancreatic ductal adenocarcinoma
PDGFRα	Platelet-derived growth factor receptor alpha
PI3K	Phosphoinositide 3-kinase
PPARγ	Peroxisome proliferator–activated receptor gamma
rhCYGB	Recombinant human cytoglobin
rhNGB	Recombinant human neuroglobin
RNS	Reactive nitrogen species
ROS	Reactive oxygen species
STAT3	Signal transducer and activator of transcription 3
TAA	Thioacetamide
TBHP	tert-Butyl hydroperoxide
TBK1	TANK-binding kinase 1
TGF-β1	Transforming growth factor beta 1
VEGF	Vascular endothelial growth factor

## References

[1] Allameh A., Niayesh-Mehr R., Aliarab A., Sebastiani G., Pantopoulos K. (2023). Oxidative Stress in Liver Pathophysiology and Disease. Antioxidants.

[2] Xing L., Tang Y., Li L., Tao X. (2023). ROS in hepatocellular carcinoma: What we know. Arch. Biochem. Biophys..

[3] Wang Z., Cao Z., Dong Y., Hong Y., Zuo L., Wang H. (2026). Hepatocyte-hepatic stellate cell interactions in liver fibrosis: Mechanisms and therapeutic implications. Hepatol. Commun..

[4] Le T.T.T., Hai H., Pham T.A., Nguyen B.T.C., Pham M.D., Hermiston M.L., Ahmad R. (2026). ROS in Liver Cancer Promotion. Reactive Oxygen Species—An Overview.

[5] Kisseleva T., Brenner D. (2021). Molecular and cellular mechanisms of liver fibrosis and its regression. Nat. Rev. Gastroenterol. Hepatol..

[6] Kawada N., Kristensen D.B., Asahina K., Nakatani K., Minamiyama Y., Seki S., Yoshizato K. (2001). Characterization of a stellate cell activation-associated protein (STAP) with peroxidase activity found in rat hepatic stellate cells. J. Biol. Chem..

[7] Thuy L.T.T., Morita T., Yoshida K., Wakasa K., Iizuka M., Ogawa T., Mori M., Sekiya Y., Momen S., Motoyama H. (2011). Promotion of liver and lung tumorigenesis in DEN-treated cytoglobin-deficient mice. Am. J. Pathol..

[8] Thi Thanh Hai N., Thuy L.T.T., Shiota A., Kadono C., Daikoku A., Hoang D.V., Dat N.Q., Sato-Matsubara M., Yoshizato K., Kawada N. (2018). Selective overexpression of cytoglobin in stellate cells attenuates thioacetamide-induced liver fibrosis in mice. Sci. Rep..

[9] Dat N.Q., Thuy L.T.T., Hieu V.N., Hai H., Hoang D.V., Thi Thanh Hai N., Thuy T.T.V., Komiya T., Rombouts K., Dong M.P. (2021). Hexa Histidine-Tagged Recombinant Human Cytoglobin Deactivates Hepatic Stellate Cells and Inhibits Liver Fibrosis by Scavenging Reactive Oxygen Species. Hepatology.

[10] Hieu V.N., Thuy L.T.T., Hai H., Dat N.Q., Hoang D.V., Hanh N.V., Phuong D.M., Hoang T.H., Sawai H., Shiro Y. (2022). Capacity of extracellular globins to reduce liver fibrosis via scavenging reactive oxygen species and promoting MMP-1 secretion. Redox Biol..

[11] Burmester T., Weich B., Reinhardt S., Hankeln T. (2000). A vertebrate globin expressed in the brain. Nature.

[12] Emara M., Turner A.R., Allalunis-Turner J. (2010). Hypoxic regulation of cytoglobin and neuroglobin expression in human normal and tumor tissues. Cancer Cell Int..

[13] Wakasugi K., Nakano T., Morishima I. (2003). Oxidized human neuroglobin acts as a heterotrimeric Galpha protein guanine nucleotide dissociation inhibitor. J. Biol. Chem..

[14] Tiso M., Tejero J., Basu S., Azarov I., Wang X., Simplaceanu V., Frizzell S., Jayaraman T., Geary L., Shapiro C. (2011). Human neuroglobin functions as a redox-regulated nitrite reductase. J. Biol. Chem..

[15] Greenberg D.A., Jin K., Khan A.A. (2008). Neuroglobin: An endogenous neuroprotectant. Curr. Opin. Pharmacol..

[16] Moschetti T., Giuffre A., Ardiccioni C., Vallone B., Modjtahedi N., Kroemer G., Brunori M. (2009). Failure of apoptosis-inducing factor to act as neuroglobin reductase. Biochem. Biophys. Res. Commun..

[17] Fiocchetti M., Fernandez V.S., Montalesi E., Marino M. (2019). Neuroglobin: A Novel Player in the Oxidative Stress Response of Cancer Cells. Oxid. Med. Cell Longev..

[18] Li W., Wu Y., Ren C., Lu Y., Gao Y., Zheng X., Zhang C. (2011). The activity of recombinant human neuroglobin as an antioxidant and free radical scavenger. Proteins Struct. Funct. Bioinf..

[19] Harrison S.A., Bedossa P., Guy C.D., Schattenberg J.M., Loomba R., Taub R., Labriola D., Moussa S.E., Neff G.W., Rinella M.E. (2024). A Phase 3, Randomized, Controlled Trial of Resmetirom in NASH with Liver Fibrosis. N. Engl. J. Med..

[20] Li W., Alazawi W., Loomba R. (2026). Current and emerging therapeutic landscape for metabolic dysfunction-associated steatohepatitis. Lancet Gastroenterol. Hepatol..

[21] Tauchen J., Huml L., Jurasek M., Regenstein J.M., Ozogul F. (2025). Synthetic and semi-synthetic antioxidants in medicine and food industry: A review. Front. Pharmacol..

[22] Garbuzenko D.V. (2022). Pathophysiological mechanisms of hepatic stellate cells activation in liver fibrosis. World J. Clin. Cases.

[23] Keppner A., Maric D., Correia M., Koay T.W., Orlando I.M.C., Vinogradov S.N., Hoogewijs D. (2020). Lessons from the post-genomic era: Globin diversity beyond oxygen binding and transport. Redox Biol..

[24] Burmester T., Ebner B., Weich B., Hankeln T. (2002). Cytoglobin: A novel globin type ubiquitously expressed invertebrate tissues. Mol. Biol. Evol..

[25] Trent J.T., Hargrove M.S. (2002). A ubiquitously expressed human hexacoordinate hemoglobin. J. Biol. Chem..

[26] Roesner A., Fuchs C., Hankeln T., Burmester T. (2005). A globin gene of ancient evolutionary origin in lower vertebrates: Evidence for two distinct globin families in animals. Mol. Biol. Evol..

[27] Fuchs C., Burmester T., Hankeln T. (2006). The amphibian globin gene repertoire as revealed by the Xenopus genome. Cytogenet. Genome Res..

[28] Kugelstadt D., Haberkamp M., Hankeln T., Burmester T. (2004). Neuroglobin, cytoglobin, and a novel, eye-specific globin from chicken. Biochem. Biophys. Res. Commun..

[29] Hoogewijs D., Ebner B., Germani F., Hoffmann F.G., Fabrizius A., Moens L., Burmester T., Dewilde S., Storz J.F., Vinogradov S.N. (2012). Androglobin: A chimeric globin in metazoans that is preferentially expressed in mammalian testes. Mol. Biol. Evol..

[30] Gorr T.A., Wichmann D., Pilarsky C., Theurillat J.P., Fabrizius A., Laufs T., Bauer T., Koslowski M., Horn S., Burmester T. (2011). Old proteins—New locations: Myoglobin, haemoglobin, neuroglobin and cytoglobin in solid tumours and cancer cells. Acta Physiol..

[31] Quinting T., Heymann A.K., Bicker A., Nauth T., Bernardini A., Hankeln T., Fandrey J., Schreiber T. (2021). Myoglobin Protects Breast Cancer Cells Due to Its ROS and NO Scavenging Properties. Front. Endocrinol..

[32] Thuy L.T.T., Van Thuy T.T., Matsumoto Y., Hai H., Ikura Y., Yoshizato K., Kawada N. (2016). Absence of cytoglobin promotes multiple organ abnormalities in aged mice. Sci. Rep..

[33] Burmester T., Hankeln T. (2014). Function and evolution of vertebrate globins. Acta Physiol..

[34] Reeder B.J. (2023). Globin Associated Oxidative Stress. Antioxidants.

[35] Reeder B.J. (2010). The redox activity of hemoglobins: From physiologic functions to pathologic mechanisms. Antioxid. Redox Signal..

[36] Sawai H., Makino M., Mizutani Y., Ohta T., Sugimoto H., Uno T., Kawada N., Yoshizato K., Kitagawa T., Shiro Y. (2005). Structural characterization of the proximal and distal histidine environment of cytoglobin and neuroglobin. Biochemistry.

[37] Hamdane D., Kiger L., Dewilde S., Green B.N., Pesce A., Uzan J., Burmester T., Hankeln T., Bolognesi M., Moens L. (2003). The redox state of the cell regulates the ligand binding affinity of human neuroglobin and cytoglobin. J. Biol. Chem..

[38] Reeder B.J., Ukeri J. (2018). Strong modulation of nitrite reductase activity of cytoglobin by disulfide bond oxidation: Implications for nitric oxide homeostasis. Nitric Oxide.

[39] Lechauve C., Chauvierre C., Dewilde S., Moens L., Green B.N., Marden M.C., Celier C., Kiger L. (2010). Cytoglobin conformations and disulfide bond formation. FEBS J..

[40] Nakatani K., Okuyama H., Shimahara Y., Saeki S., Kim D.H., Nakajima Y., Seki S., Kawada N., Yoshizato K. (2004). Cytoglobin/STAP, its unique localization in splanchnic fibroblast-like cells and function in organ fibrogenesis. Lab. Investig..

[41] Hamdane D., Kiger L., Dewilde S., Green B.N., Pesce A., Uzan J., Burmester T., Hankeln T., Bolognesi M., Moens L. (2004). Coupling of the heme and an internal disulfide bond in human neuroglobin. Micron.

[42] Reeder B.J. (2023). Insights into the function of cytoglobin. Biochem. Soc. Trans..

[43] Tanaka Y., Sato-Matsubara M., Tsuruta D., Tanaka H., Kadono C., Sugawara K., Kawada N., Wakamatsu K., Ito S., Yoshizato K. (2024). Cytoglobin functions as a redox regulator of melanogenesis in normal epidermal melanocytes. Pigm. Cell Melanoma Res..

[44] Wilson M.T., Reeder B.J. (2022). The peroxidatic activities of Myoglobin and Hemoglobin, their pathological consequences and possible medical interventions. Mol. Asp. Med..

[45] Gardner P.R. (2005). Nitric oxide dioxygenase function and mechanism of flavohemoglobin, hemoglobin, myoglobin and their associated reductases. J. Inorg. Biochem..

[46] Jayaraman T., Tejero J., Chen B.B., Blood A.B., Frizzell S., Shapiro C., Tiso M., Hood B.L., Wang X., Zhao X. (2011). 14-3-3 binding and phosphorylation of neuroglobin during hypoxia modulate six-to-five heme pocket coordination and rate of nitrite reduction to nitric oxide. J. Biol. Chem..

[47] Wang X., Liu J., Zhu H., Tejima E., Tsuji K., Murata Y., Atochin D.N., Huang P.L., Zhang C., Lo E.H. (2008). Effects of neuroglobin overexpression on acute brain injury and long-term outcomes after focal cerebral ischemia. Stroke.

[48] Thuy L.T.T., Matsumoto Y., Thuy T.T.V., Hai H., Suoh M., Urahara Y., Motoyama H., Fujii H., Tamori A., Kubo S. (2015). Cytoglobin deficiency promotes liver cancer development from hepatosteatosis through activation of the oxidative stress pathway. Am. J. Pathol..

[49] Liu X., El-Mahdy M.A., Boslett J., Varadharaj S., Hemann C., Abdelghany T.M., Ismail R.S., Little S.C., Zhou D., Thuy L.T.T. (2017). Cytoglobin regulates blood pressure and vascular tone through nitric oxide metabolism in the vascular wall. Nat. Commun..

[50] Okina Y., Sato-Matsubara M., Matsubara T., Daikoku A., Longato L., Rombouts K., Thanh Thuy L.T., Ichikawa H., Minamiyama Y., Kadota M. (2020). TGF-beta1-driven reduction of cytoglobin leads to oxidative DNA damage in stellate cells during non-alcoholic steatohepatitis. J. Hepatol..

[51] Li Z., Wei W., Chen B., Cai G., Li X., Wang P., Tang J., Dong W. (2016). The Effect of rhCygb on CCl4-Induced Hepatic Fibrogenesis in Rat. Sci. Rep..

[52] Van Thuy T.T., Thuy L.T., Yoshizato K., Kawada N. (2017). Possible Involvement of Nitric Oxide in Enhanced Liver Injury and Fibrogenesis during Cholestasis in Cytoglobin-deficient Mice. Sci. Rep..

[53] Thuy L.T.T., Hai H., Kawada N., Albano E., Parola M. (2015). Antioxidant Approach to the Therapy of Chronic Liver Diseases. Studies on Hepatic Disorders.

[54] Zhao W., Zhuang P., Chen Y., Wu Y., Zhong M., Lun Y. (2023). “Double-edged sword” effect of reactive oxygen species (ROS) in tumor development and carcinogenesis. Physiol. Res..

[55] Zhang J., Pei Y., Yang W., Yang W., Chen B., Zhao X., Long S. (2019). Cytoglobin ameliorates the stemness of hepatocellular carcinoma via coupling oxidative-nitrosative stress signals. Mol. Carcinog..

[56] Yang S., Cai M., Yang Y., Huang Y., Hou T., Zhang J. (2025). Cytoglobin suppresses oxidative damage and compensatory proliferation via inhibiting AKT/ERK1/2/CyclinD1 axis in hepatocellular carcinoma. Am. J. Cancer Res..

[57] Loomba R., Friedman S.L., Shulman G.I. (2021). Mechanisms and disease consequences of nonalcoholic fatty liver disease. Cell.

[58] Zhang Y., Ren L., Tian Y., Guo X., Wei F., Zhang Y. (2024). Signaling pathways that activate hepatic stellate cells during liver fibrosis. Front. Med..

[59] Motoyama H., Komiya T., Thuy L.T.T., Tamori A., Enomoto M., Morikawa H., Iwai S., Uchida-Kobayashi S., Fujii H., Hagihara A. (2014). Cytoglobin is expressed in hepatic stellate cells, but not in myofibroblasts, in normal and fibrotic human liver. Lab. Investig..

[60] Hoang D.V., Thuy L.T.T., Hai H., Hieu V.N., Kimura K., Oikawa D., Ikura Y., Dat N.Q., Hoang T.H., Sato-Matsubara M. (2022). Cytoglobin attenuates pancreatic cancer growth via scavenging reactive oxygen species. Oncogenesis.

[61] Zou Z., Yu Q., Yang Y., Wang F., Zhu P., Zhang X., Zhang J. (2024). Cytoglobin attenuates melanoma malignancy but protects melanoma cells from ferroptosis. Mol. Med. Rep..

[62] Fan C., Luo Z., Zheng Q., Xu Y., Xu Y., Chen J., Meng Y., Jiang H., Liu K., Xi Y. (2025). Cytoglobin augments ferroptosis through autophagic degradation of ferritin in colorectal cancer cells. Mol. Cell Biochem..

[63] Feng Y., Wu M., Li S., He X., Tang J., Peng W., Zeng B., Deng C., Ren G., Xiang T. (2018). The epigenetically downregulated factor CYGB suppresses breast cancer through inhibition of glucose metabolism. J. Exp. Clin. Cancer Res..

[64] Shivapurkar N., Stastny V., Okumura N., Girard L., Xie Y., Prinsen C., Thunnissen F.B., Wistuba I.I., Czerniak B., Frenkel E. (2008). Cytoglobin, the newest member of the globin family, functions as a tumor suppressor gene. Cancer Res..

[65] Jourd’heuil F., Mathai C., Cat Pham L.G., Gilliard K., Balnis J., Overmyer K.A., Coon J.J., Jaitovich A., Boivin B., Jourd’heuil D. (2025). Cytoglobin scavenges intracellular hydrogen peroxide and regulates redox signals in the vasculature. Redox Biol..

[66] De Backer J., Hoogewijs D. (2024). The cytoglobin-dependent transcriptome in melanoma indicates a protective function associated with oxidative stress, inflammation and cancer-associated pathways. Sci. Rep..

[67] Sun Y., Jin K., Mao X.O., Zhu Y., Greenberg D.A. (2001). Neuroglobin is up-regulated by and protects neurons from hypoxic-ischemic injury. Proc. Natl. Acad. Sci. USA.

[68] Reuss S., Saaler-Reinhardt S., Weich B., Wystub S., Reuss M.H., Burmester T., Hankeln T. (2002). Expression analysis of neuroglobin mRNA in rodent tissues. Neuroscience.

[69] Cai B., Li W., Mao X., Winters A., Ryou M.G., Liu R., Greenberg D.A., Wang N., Jin K., Yang S.H. (2016). Neuroglobin Overexpression Inhibits AMPK Signaling and Promotes Cell Anabolism. Mol. Neurobiol..

[70] Wu F., He J., Deng Q., Chen J., Peng M., Xiao J., Zeng Y., Yi L., Li Z., Tian R. (2023). Neuroglobin inhibits pancreatic cancer proliferation and metastasis by targeting the GNAI1/EGFR/AKT/ERK signaling axis. Biochem. Biophys. Res. Commun..

[71] Li L., Zhang H., Li W., Yuan Z., Zhao W., Luo Z. (2025). Neuroglobin-promoted autophagy inhibits the proliferation, migration and invasion of human glioblastoma cells through the EGFR/PI3K/AKT/mTOR signaling pathway. J. Transl. Med..

[72] Fordel E., Thijs L., Moens L., Dewilde S. (2007). Neuroglobin and cytoglobin expression in mice. Evidence for a correlation with reactive oxygen species scavenging. FEBS J..

[73] Okogwu O.I., Xie P., Zhao Y., Fan H. (2014). Organ-dependent response in antioxidants, myoglobin and neuroglobin in goldfish (Carassius auratus) exposed to MC-RR under varying oxygen level. Chemosphere.

[74] Zhang J., Lan S.J., Liu Q.R., Liu J.M., Chen X.Q. (2013). Neuroglobin, a novel intracellular hexa-coordinated globin, functions as a tumor suppressor in hepatocellular carcinoma via Raf/MAPK/Erk. Mol. Pharmacol..

[75] Liang X., Wen Y., Feng C., Xu L., Xian Y., Xie H., Huang J., Huang Y., Zhao X., Gao X. (2024). Neuroglobin protects dopaminergic neurons in a Parkinson’s cell model by interacting with mitochondrial complex NDUFA10. Neuroscience.

[76] Gao W., Mo C., Feng W., Pan X., Qin H. (2023). Research on the Effects of Neuroglobin on Ferroptosis in the Nerve Cells. Chin. Med. Nat. Prod..

[77] Xiang Q., Zhou D., Xiang X., Le X., Deng C., Sun R., Li C., Pang H., He J., Zheng Z. (2023). Neuroglobin plays as tumor suppressor by disrupting the stability of GPR35 in colorectal cancer. Clin. Epigenetics.

[78] Kim Y., Kim M., Kim S.D., Yoon N., Wang X., Bae G.U., Song Y.S. (2022). Distribution of Neuroglobin in Pericytes is Associated with Blood-Brain Barrier Leakage against Cerebral Ischemia in Mice. Exp. Neurobiol..

[79] De Simone G., Sbardella D., Oddone F., Pesce A., Coletta M., Ascenzi P. (2021). Structural and (Pseudo-)Enzymatic Properties of Neuroglobin: Its Possible Role in Neuroprotection. Cells.

[80] Forman H.J., Zhang H. (2021). Targeting oxidative stress in disease: Promise and limitations of antioxidant therapy. Nat. Rev. Drug Discov..

[81] Ebrahimi S.B., Samanta D. (2023). Engineering protein-based therapeutics through structural and chemical design. Nat. Commun..

[82] Niazi S.K., Magoola M. (2023). mRNA and Synthesis-Based Therapeutic Proteins: A Non-Recombinant Affordable Option. Biologics.

[83] Jain S., Gupta S., Patiyal S., Raghava G.P.S. (2024). THPdb2: Compilation of FDA approved therapeutic peptides and proteins. Drug Discov. Today.

[84] Liu M., Svirskis D., Proft T., Loh J., Yin N., Li H., Li D., Zhou Y., Chen S., Song L. (2025). Progress in peptide and protein therapeutics: Challenges and strategies. Acta Pharm. Sin. B.

[85] Seo J., Oh D.B. (2022). Mannose-6-phosphate glycan for lysosomal targeting: Various applications from enzyme replacement therapy to lysosome-targeting chimeras. Anim. Cells Syst..

[86] Pathivada K., Glassman P.M. (2025). Half-life extension of therapeutics: Applications and mechanisms. J. Pharmacol. Exp. Ther..

[87] Hamdane D., Kiger L., Dewilde S., Uzan J., Burmester T., Hankeln T., Moens L., Marden M.C. (2005). Hyperthermal stability of neuroglobin and cytoglobin. FEBS J..

[88] Diaz-Manera J., Kishnani P.S., Kushlaf H., Ladha S., Mozaffar T., Straub V., Toscano A., van der Ploeg A.T., Berger K.I., Clemens P.R. (2021). Safety and efficacy of avalglucosidase alfa versus alglucosidase alfa in patients with late-onset Pompe disease (COMET): A phase 3, randomised, multicentre trial. Lancet Neurol..

[89] Yadav D., Dewangan H.K. (2021). PEGYLATION: An important approach for novel drug delivery system. J. Biomater. Sci. Polym. Ed..

[90] Liebner R., Altinoglu S., Selzer T. (2022). A Road Map to GMP Readiness for Protein Therapeutics—Drug Product Process Development for Clinical Supply. J. Pharm. Sci..

[91] Mangia A., Valenti L.V.C. (2025). Safety Choice Drivers of the Coming Treatment Options for Non-Cirrhotic Metabolic Steatohepatitis. Liver Int..

